# Heat Strain, External Workload, and Chronic Kidney Disease in Tropical Settings: Are Endurance Athletes Exposed?

**DOI:** 10.3389/fphys.2019.01403

**Published:** 2019-11-21

**Authors:** Daniel Rojas-Valverde, Guillermo Olcina, Randall Gutiérrez-Vargas, Jennifer Crowe

**Affiliations:** ^1^Centro de Investigación y Diagnóstico en Salud y Deporte, Escuela Ciencias del Movimiento Humano y Calidad de Vida, Universidad Nacional, Heredia, Costa Rica; ^2^Grupo en Avances en el Entrenamiento Deportivo y Acondicionamiento Físico (GAEDAF), Facultad Ciencias del Deporte, Universidad de Extremadura, Cáceres, Spain; ^3^Instituto Regional de Estudios en Sustancias Tóxicas (IRET), Universidad Nacional, Heredia, Costa Rica

**Keywords:** sport, physical endurance, heat strain, acute kidney injury, running

## Introduction

Tropical regions are currently facing a great challenge regarding very high prevalence of chronic kidney disease (CKD). In recent years, a condition called chronic kidney disease of unknown etiology (CKDu), also called CKD of non-traditional origin (CKDnt) or Mesoamerican Nephropathy has been reported (Wesseling et al., 2013, 2015; Wegman et al., 2015). This renal condition has been clearly documented in several so-called hot spots around the globe, typically in low-altitude communities near the western coast of the American continent. Though CKDu presents as typical CKD (Jha et al., 2013), CKDu patients do not present typical risk factors such as obesity, advanced age, hypertension, or diabetes. Instead, they tend to be young, otherwise healthy individuals that often live and work where dehydration and high internal and external physical loads are common. Although the etiology of the disease remains unclear (González-Quiroz et al., 2018; Chapman et al., 2019; Pearce and Caplin, 2019), most researchers agree that CKDu etiology is likely multifactorial and that chronic heat exposure, high external workload, and dehydration are associated with the disease (Wegman et al., 2015; Kupferman et al., 2018). Until now, CKDu in Mesoamerica has been studied mostly in agricultural populations (Crowe et al., 2013, 2015; García-Trabanino et al., 2015; Laws et al., 2016; Wesseling et al., 2016; Butler-Dawson et al., 2018; Kupferman et al., 2018).

Recently, the possibility of elevated rates of CKD in other populations exposed to high external heat and heavy internal heat loads from work/exercise has been raised. These populations could include endurance athletes (Eichner, 2017) in sports such as running, cycling, triathlon, open swimming, adventure races, and other long-duration disciplines. Such athletes usually undertake tremendous physical effort under high heat and humidity during a large number of hours and even consecutive days in multi-stage events in tropical settings (Gutiérrez-Vargas et al., 2018; McDermott et al., 2018; Rojas-Valverde, 2019). Although there are clear differences between CKDu occupational populations and athletes including socio-economic levels, non-optimal working conditions, poor access to health services, lack of recovery time, and low educational levels, there are notable environmental and contextual similarities between both populations.

Since people living and working in agricultural communities often face a wide range of social-economic determinants of health and are exposed to wide variety agents that could feasibly be on the etiologic pathway to CKDu, methodologies to study the effect of heat load on the kidney are complex. The athletes who participate in endurance events represent a privileged sample that could allow the isolation of factors that lead to CKD and provide insights into how external workload, dehydration and heat strain could contribute to the development of adverse renal conditions in athletes as well as populations in more vulnerable populations.

**Figure 1:**
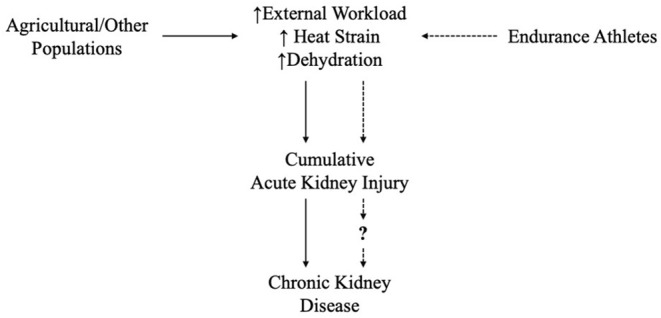
Chronic Kidney Disease common risk factors between agricultural populations and endurance athletes in tropical settings. Dotted line flow needs more longitudinal research and evidence.

The existing evidence between heat exposure and CKDu in addition to imminent changes in temperature due to global warming have already prompted the question of whether athletes might also be at risk for kidney damage (Eichner, 2017). The sum of external workload and internal factors leading to thermal strain is of great concern, particularly when environmental temperature exceeds normal body temperature. The effect of high thermal stress (e.g., rise in internal temperature, heat loss restriction due to high humidity, and other factors) on the health and performance of athletes has been previously reported (Che Muhamed et al., 2016; Gutiérrez-Vargas et al., 2018; McDermott et al., 2018; Omassoli et al., 2019). Although these conditions are similar to those presented by populations in which CKDu has been diagnosed (see Figure 1). AKI leading to CKD has not been directly related to sports practice. However, there is evidence about increasing cases of acute kidney injury (AKI) in endurance sports related to external workload, heat and dehydration (Junglee et al., 2013; Hou et al., 2015; Kao et al., 2015; Bongers et al., 2018), although there remains a lack of information about whether AKI in sports could lead to CKD.

Recently, it has been shown that the practice of endurance sports can cause exertional rhabdomyolysis conditions (Hoffman et al., 2012; Kim et al., 2015), and that this can trigger transitional AKI (Boulter et al., 2011; Chlíbková et al., 2015; Hoffman and Weiss, 2016) due to the release of sarcoplasmic proteins into the bloodstream as a consequence of damage and disintegration of striated muscle during strenuous physical exertion (Bosch et al., 2009; Tietze and Borchers, 2014; Olcina et al., 2018). The preferred biomarkers to diagnose both conditions are serum creatinine and cystatin C levels for kidney function and creatine kinase and lactate dehydrogenase for muscle damage. According to the RIFLE (Risk, Injury, Failure, Loss of Kidney Function, and End-stage kidney disease) categorization, a risk of renal injury exists when serum creatinine (S-Cr) increases 1.5 times; a lesion when S-Cr 2 times, and failure when the S-Cr increases 3 times or values >4 mg/dL (Bellomo et al., 2004). Another classification system, the Acute Kidney Injury Network (AKIN) classification considers AKI to occur when at least one of the following conditions are met in the last 48 h: (a) absolute increase of ≥0.3 mg/dL, (b) increase of 1.5 times above the baseline, or (3) oliguria (urination <0.5 mL/kg per hour per >6 h) (Lopes and Jorge, 2013). In addition, other biomarkers have been recently proposed in order to differentiate functional and subclinical AKI as: cystatin-C, serum albumin, neutrophil gelatinase-associated lipocalin, and kidney injury molecule 1 (McCullough et al., 2013).

Athletes experiencing AKI according to these definitions have been documented to regain baseline renal function in a matter of 1–15 days (Kim et al., 2015; Abbas et al., 2019; Rojas-Valverde et al., 2019), however, documentation also exists of more serious consequences, leading to the death of the athlete in combination to other potential risks (Asserraji et al., 2014). Due to the lack of information about whether the repeated AKI can lead to future CKD (Hoffman and Weiss, 2016), there is a need for studies that shed light on the potential of the combination of environment and physical thermal load in athletes to trigger future CKD, especially in tropical settings, where conditions of high thermal stress exist almost year-round in some regions (Gutiérrez-Vargas et al., 2017, 2018). Unfortunately, in order to observe whether AKI caused by heat exposure and prolonged physical exercise provokes CKD on the long-term requires cohort studies in a long timeframe; such a timeframe could be too late for affected athletes (Eichner, 2017). Other contextual factors that could influence AKI incidence should be explored as internal thermal load indicators, slope variations, age, finish time, carried weight during running, dehydration status and other contextual variables.

## Discussion

Existing evidence regarding AKI in hospitalized and occupational (working) populations, as well as the similarities in thermal load experienced by CKDu-affected occupational populations and athlete populations in the tropics, beg the question of whether athletes in hot environments might be experiencing AKI and eventually CKD. Currently available technological tools and methods allow to objectively study cases of AKI and CKD, improving parameters for accurate diagnosis (Clarkson et al., 2006; Stahl et al., 2019) and new markers and methods have been used for their identification (Colombini et al., 2012; McCullough et al., 2013).

Enough is known about the potential risk that we must call on authorities, including universities, ministries, federations, sports committees and other involved institutions, to study, and take preventative regulatory actions in the tropical and Mesoamerican region to protect athletes who compete for prolonged periods at high levels of thermal stress. This is of particular urgency given the recent increase in popularity and quantity of this type of event and the fact that there is no regional and organizational platform in place to insure implementation of endurance events in a secure manner. Regulation of aspects such as the time of events, heat-exposure, stricter participation criteria, regular medical check-ups, among others, are essential to avoid a regional health problem related to physical exercise.

Through this call for attention, the regional and international scientific community is urged not to wait for the first cases of CKD in athletes related to the combination of three above mentioned factors: heat strain, dehydration, and high external workload, to be documented before acting, since preliminary evidence (i.e., frequent reports of exertional rhabdomyolysis, AKI) are more than enough to demonstrate the need to take timely measures. Since cumulative AKI events have been shown to make individuals more likely to develop CKD in the future (Heung et al., 2016; Hsu and Hsu, 2016), it is highly probable that the same phenomenon is occurring in athletes.

Endurance athletes in tropical regions are exposed to conditions (heat strain, dehydration, and high external workload) similar to those experienced by working populations known to suffer from AKI and CKDu (Gutiérrez-Vargas et al., 2018; Rojas-Valverde et al., 2019), which is why attention should be paid to this factor as a determining point for the monitoring and treatment of this condition in endurance athletes who they carry out long-term activities at moderate to high intensities. We therefore advocate more research in athlete populations in tropical settings in order to (1) protect athletes who may be exposed under the current lack of regulation and (2) provide possible mechanistic insights that might help understand and intervene with CKDu-affected working populations.

## Author Contributions

DR-V and JC contributed to the conceptualization, the preparation, and the writing of the original draft. DR-V contributed to the literature search. JC, GO, and RG-V critically revised the manuscript and contributed to the supervision. DR-V, JC, GO, and RG-V contributed to the final manuscript approval.

### Conflict of Interest

The authors declare that the research was conducted in the absence of any commercial or financial relationships that could be construed as a potential conflict of interest.
